# Comparison of the Efficacy of Platelet-Rich Plasma (PRP) and Local Corticosteroid Injection in Periarthritis Shoulder: A Prospective, Randomized, Open, Blinded End-Point (PROBE) Study

**DOI:** 10.7759/cureus.29253

**Published:** 2022-09-17

**Authors:** Govind K Gupta, Shubhendu Shekhar, Zeya Ul Haque, Subhajit Halder, Amit K Manjhi, Arpita Rai

**Affiliations:** 1 Orthopedics, Rajendra Institute of Medical Sciences, Ranchi, IND; 2 Oral Medicine and Radiology, Rajendra Institute of Medical Sciences, Ranchi, IND; 3 Orthopedics, Mahatma Gandhi Memorial Medical College and Hospital, Jamshedpur, IND

**Keywords:** intra-articular injection, triamcinolone, platelet-rich plasma (prp), steroid, periarthritis

## Abstract

Background

Periarthritis or frozen shoulder, also called adhesive capsulitis, is characterized by stiffness and pain along with gradual loss of active and passive movement in the glenohumeral joint. More than 2-5% of the population suffers from periarthritis with a higher incidence in the age group of 40-60 years. The various treatment modalities used for its management include simple physiotherapy, short-wave therapy, ultrasonic therapy, transcutaneous electrical nerve stimulation, hydrotherapy, analgesics, intra-articular injections, manipulation under general anesthesia (MUA), and surgical management. The application of intra-articular steroid injection has been a common and efficacious option in rapidly diminishing shoulder pain and disability. Some recent studies reported a better outcome using platelet-rich plasma (PRP) injections in frozen shoulder cases. Hence, this randomized controlled trial was conducted to compare the efficacy of intra-articular injections of PRP and triamcinolone in patients of shoulder periarthritis in a population from the eastern region of India

Methodology

A total of 60 patients with periarthritis shoulder were allocated into two groups after randomization. Group A received 2 mL autologous PRP, and Group B received 2 mL of triamcinolone (40 mg/mL) intra-articular injection. Patients were followed up on the 4th week, 12th week, and 24th week. The assessment of pain and function using the visual analog scale (VAS) score and the Disabilities of Arm, Shoulder, and Hand (DASH) score, respectively, was done at each follow-up. The primary analyses of both primary and secondary outcomes were conducted in the intention-to-treat (ITT) population. SPSS version 24 (IBM Corp., Armonk, NY, USA) was used for data analysis.

Results

The mean VAS score in the PRP and triamcinolone groups was 14.33 ± 3.79 and 31.63 ± 7.62, respectively (p = 0.0001) after 24 weeks. The mean DASH score in the PRP and triamcinolone groups was 18.08 ± 8.08 and 31.76 ± 3.63, respectively (p = 0.0001), which shows significant improvement in both pain and disability scores in the PRP group after 24 weeks.

Conclusions

The triamcinolone group showed better short-term outcomes whereas PRP showed better long-term outcomes in reducing pain and disability scores in terms of VAS and DASH scores.

## Introduction

Periarthritis of the shoulder is characterized by functional loss of passive and active shoulder motion. This condition was termed by Duplay in 1896 and later substituted by the term frozen shoulder by Codman in 1932. Subsequently, Nevaiser introduced the term adhesive capsulitis [1]. This disorder is defined by the American Shoulder and Elbow Surgeons as a condition of significant restriction of both active and passive motion of the shoulder joints because of an unknown etiology that occurs without an intrinsic shoulder disorder [2].

The definite pathophysiology of periarthritis remains unclear. The progressive fibrosis causing the contracture of the glenohumeral joint capsule results in pain and stiffness [3]. Periarthritis can be primary or secondary. The primary (or idiopathic) type occurs without any known trauma or provoking event. The secondary type is often observed after periarticular trauma, fracture, or dislocation of the glenohumeral joint [4].

According to recent studies, the incidence of periarthritis is 2-5% in the general population [5,6]. The affected population includes 70% females. The idiopathic type often involves the non-dominant extremity, while 40-50% of cases have been reported as bilateral involvement. Regardless of the etiology, the condition is more prevalent in the 40-60-year age group [4,7]. The risk factors for developing periarthritis include diabetes. Patients with type I diabetes have a 40% chance of developing periarthritis. Up to 29% of individuals with type II diabetes may develop this condition. Thyroid disease, Parkinson’s disease, cardiac disease, autoimmune disease, chronic obstructive pulmonary disorder, and myocardial infarction are also linked with increased incidence of periarthritis or adhesive capsulitis [3,8].

In most cases, periarthritis resolves spontaneously or it can last for up to three years [9]. Various treatment approaches have been used and explored to treat this disorder. Physical therapy individually or in combination with short-wave therapy, ultrasonic therapy, transcutaneous electrical nerve stimulation, and hydrotherapy is used [10]. Pharmacological treatment includes the use of analgesic or non-steroidal anti-inflammatory drugs, oral or intra-articular use of corticosteroids, and sodium hyaluronate injections. Other approaches to treat periarthritis include manipulation under anesthesia (MUA), dilation or distension of the capsule, and arthroscopic or open capsular release (arthroscopic capsulotomy) [3,4,11].

Platelet-rich plasma (PRP) is an emerging entity in the field of tissue engineering and regenerative medicine due to its availability, affordability, and minimally invasive procedure. Its autologous nature prevents an immunological reaction and offers good therapeutic safety. Recently, evidence in immune-mediated disorders and inflammatory processes has garnered attention due to their anti-inflammatory effects through the inhibition of nuclear factor kappa B signaling in target cells and by tissue inhibitor of matrix metalloproteinase. The creation and remodeling of the extracellular matrix also encompass a function of platelet growth factors which further supports this treatment modality [12]. The application of intra-articular steroid injection has been a common and efficacious option in rapidly diminishing shoulder pain and disability [5]. Some recent studies show a better outcome using PRP injections in frozen shoulder cases [13]. A systemic review and meta-analysis by Sun et al. described that patients taking a single steroid injection for a frozen shoulder is effective and safe and improves functional outcomes and pain scores [14].

Corticosteroid injections have been associated with prominent side effects, which have led to the conception of modalities such as PRP. This randomized trial aimed to evaluate and compare the efficacy of intra-articular injections of PRP and steroid (triamcinolone) in periarthritis. We hypothesized that PRP would prove more effective in relieving pain and improving function. Several studies have reported comparative analyses of steroids and PRP. Most of these were conducted outside India. Studies by Upadhyay et al. [15], Kothari et al. [16], and Kumar et al. [17] reported the effect of PRP versus steroids in periarthritis in the Indian population. One study from the eastern part of India with a similar intervention was conducted by Barman et al. [18], but the follow-up period was only 12 weeks. Hence, this study was conducted to analyze the comparative efficacy of PRP versus steroids in periarthritis with a follow-up duration of 24 weeks in a population from the eastern region of India.

## Materials and methods

Trial design

This study was a parallel-group, prospective, randomized, open, blinded end-point (PROBE), single-center clinical study. Randomization was done in permuted blocks of varying sizes (2, 4, 6) using a sealed envelope website (computer-generated) [19]. There was central randomization, and the person doing randomization was not part of the study. The investigator assigning intervention telephonically contacted the randomizer on the recruitment of every new patient regarding the group to which the patient was assigned. Another investigator (other than the one assigning intervention) assessed the outcomes of the patients without any knowledge of the study group to which the patient belonged to. Patients were recruited to different treatment regimens following proper randomization. Unlike double-blind studies, the treatment regimens were recognizable to both physicians and patients. The trial was conducted according to the principles of the Consolidated Standards of Reporting Trials (CONSORT).

Site of the study

The study was conducted from December 2020 to December 2021 at the Department of Orthopaedics, Rajendra Institute of Medical Sciences (RIMS), Ranchi Jharkhand, India. Ethical approval was obtained (vide reference number: 123, dated November 23, 2020) from the Institutional Ethical Committee of RIMS, Ranchi.

Participants

A total of 60 patients from the outpatient department (OPD), Department of Orthopedics, RIMS who were clinically diagnosed to have periarthritis shoulder and willing to participate were randomized into two groups. A written informed consent regarding participation was obtained before recruitment. The complete procedure of the study was explained to all participants in their language by the investigator before recruitment. The inclusion and exclusion criteria are presented in Table 1 and Table 2, respectively.

**Table 1:** Inclusion criteria.

Serial number	Criteria
1	Patients aged between 30 and 75 years
2	Patients having shoulder pain for at least one month and associated with more than one-third of loss of active shoulder flexion, abduction, and external rotation
3	A normal anteroposterior radiograph of the glenohumeral joint in neutral rotation
4	Willingness to refrain from any other auxiliary treatment modality

**Table 2:** Exclusion criteria.

Serial number	Criteria
1	Patients with any previous treatment in the form of local injections
2	Suffering from symptoms of shoulder pain due to other reasons
3	Unwillingness to participate in the study
4	Any intrinsic glenohumeral pathology
5	History of shoulder trauma/surgery, and clinical evidence of complex regional pain syndrome
6	History of injection in the involved shoulder joint during the preceding six months
7	Non-steroidal anti-inflammatory drugs intake in the last seven days
8	Patients with hematological disorders or on antiplatelet or anticoagulant therapy
9	Patients with thyroid disorders, pulmonary disorders particularly emphysema and chronic bronchitis, neoplastic disorders
10	Pregnant or breastfeeding females

Sample size

The sample size was calculated by OpenEpi, Version 3, an open-source calculator based on the findings of the study by Kothari et al., in which the mean VAS score for PRP and steroid group were reported [16]. The calculated sample size was 29 for each group (Table 3). Rounding off to the nearest, the total sample size was finally set as 60 (30 per group).

**Table 3:** Sample size calculation. *Difference between the means.

	Group 1	Group 2	Difference*
Mean	1.9	3.4	-1.5
Standard deviation	1.8	2.2	
Variance	3.24	4.84	
Sample size of Group 1	29		
Sample size of Group 2	29		
Total sample size	58		
Confidence interval (two-sided)	95%		
Power	80		
Ratio of sample size (Group 2/Group 1)	1		

Procedure

All information about the history, clinical features, examination findings, and treatment (if any were taken before) were recorded in a predesigned proforma. All patients were subjected to routine blood investigation and radiographic examinations of the cervical spine and ipsilateral shoulder under study.

Before administrating the injection, povidone-iodine and ethyl alcohol were applied to the skin. One milliliter of 2% lignocaine with adrenaline was injected at the injection site after administering the test dose. After 10 minutes, the proposed injection was injected. If any resistance was felt during the injection, the needle was withdrawn slightly and again injected.

The first group of patients was administered 2 mL of triamcinolone (40 mg/mL). The second group was given 2 mL autologous PRP. To prepare PRP, about 15 mL of the patient’s blood was drawn through a scalp vein catheter. The PRP was prepared using a differential centrifugation technique with two spins. The blood was collected in three citrate tubes having 0.9% sodium citrate as an anticoagulant. The first spin was performed at 1,500 rpm for 15 minutes using a laboratory centrifuge. This spin separated the red blood cells from the rest of the components. The upper half of the supernatant was discarded. The lower halves of the supernatant from all three tubes were transferred into another plain tube for the second spin. The second spin was performed at 2,500 rpm for 10 minutes. The upper half of the supernatant was discarded. Three milliliters of the lower half was taken into a syringe having 0.1 mL of calcium chloride. At the end of the preparation of PRP, 1 mL of obtained PRP (as a sample) was sent for platelet count, and the count was compared with the patient’s platelet count. Another 2 mL was used for intra-articular injection. The platelet count in the PRP preparation was 860,000 ± 74,500 platelets per mm^3^ which were 4.2 ± 1.37 times higher than whole blood values. In our study, we injected freshly prepared PRP (within 30 minutes of preparation), as a study by Blajchman [20] reported that platelets may alter the shape and reduce the functional properties, including the degranulation of α-granules due to prolonged storage.

All patients were advised regarding post-injection care. The possibility of pain increasing during the initial two weeks was explained to the patient. Post-injection, patients were prescribed paracetamol (650 mg BD orally for five days) for pain relief in both groups. Patients were advised to rest during the initial two weeks and avoid strenuous activities by the extremity under study after the injection. Physiotherapy was advised for both groups. Bilateral cases were injected simultaneously, and the post-injection protocol was the same.

Assessment and follow-up

After inclusion in the study, demographic data, baseline clinical findings, duration of pain, dominancy of the affected side, and associated comorbidities were recorded. Any relevant X-ray findings were noted. Special investigations were performed as per comorbidity present in a case. The follow-ups were done in the 4th week, 12th week, and 24th week for all patients of both groups. The assessment of pain and function through the VAS and the Disabilities of Arm, Shoulder, and Hand (DASH) score, respectively, was done at each follow-up. Any adverse effects were noted and reported. All data were documented in case report form (CRF) designed for the project and in Excel sheets for analysis.

Outcome measures

The primary outcome of the study was the pain reduction assessed using the VAS after the injections. The DASH scores were assessed as a secondary outcome.

Statistical analysis

The primary analyses of both primary and secondary outcomes were conducted in the intention-to-treat (ITT) population (i.e., all randomized participants for whom consent was given to use data). SPSS version 24 (IBM Corp., Armonk, NY, USA) was used for data analysis. The data with categorical variables were expressed as numbers and percentages, while the continuous variables were expressed as the mean ± standard deviation (SD). An unpaired t-test was used for analyzing continuous variables in the intergroup analysis. The Fisher’s exact test and Pearson’s chi-square test were used for analyzing categorical variables. P-values of <0.05 were considered to be significant.

## Results

**Figure 1:**
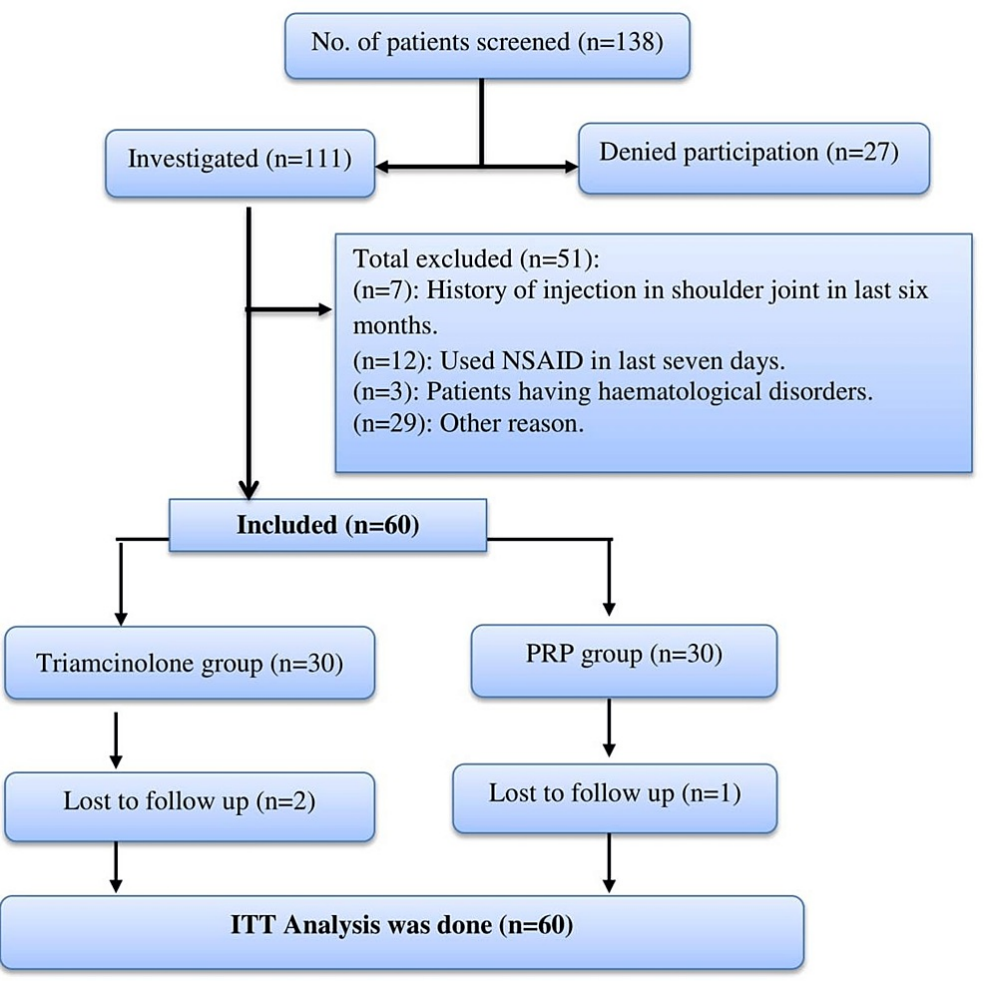
CONSORT diagram. n: number of patients; ITT: intention to treat; NSAIDs: non-steroidal anti-inflammatory drugs

A total of 60 patients were recruited for the study and randomized equally into two groups. One patient from the PRP group and two patients from the triamcinolone group did not come for the last follow-up (24 weeks). However, analyses were done for a total of 60 patients as per the ITT analysis protocol (See Figure 1).

The demographic data presented in Table 4 reveals that both groups were similar in characteristics. There was no significant difference between both groups in the baseline characteristics, e.g., age, gender, the dominance of the affected side, duration of symptoms, and presence of diabetes mellitus. This revealed that patient variability was not present between both groups. Moreover, the inclusion and exclusion criteria were followed strictly during patient recruitment and randomization. Therefore, the possibility of patient variability in the study groups was negligible.

**Table 4:** Clinicodemographic characteristics. #: Unpaired t-test was used for intragroup analysis; a: Fisher’s exact test/Pearson’s chi-square were used. PRP: platelet-rich plasma; SD: standard deviation

Variables	Triamcinolone (n = 30)	PRP (n = 30)	P-value
Age (mean ± SD)	46.70 ± 7.13	47.8 ± 9.56	0.615^#^
Sex (n)			
Male	13	12	0.793^a^
Female	17	18	
Involved side (n)			
Dominant	12	10	0.592^a^
Non-dominant	18	20	
Duration of symptoms in months (mean ± SD)	3.217 ± 0.887	3.567 ± 1.015	0.160^#^
History of diabetes mellitus (n)			
Present	14	13	0.7952^a^
Absent	16	17	

The patients with frozen shoulders were aged from 33 to 67 years. The incidence of the disease was higher in the fifth decade of life (46.67%). The mean age of the patients was 47.25 ± 8.38 years (in triamcinolone and PRP treatment groups). The incidence of the disease was higher in females (58.33%) compared to males (41.67%). In the triamcinolone group, there were 56.67% females, while in the PRP group, there were 60% females.

Among 60 patients, 30 received prolotherapy (PRP injection) and 30 received triamcinolone injection for frozen shoulder. Table 5 represents the outcome analysis of both groups. In the first follow-up (four weeks), the mean VAS score in the triamcinolone group was 46.27 ± 8.17 while it was in 51.70 ± 6.02 in the PRP group. This significantly shows better improvement of pain with triamcinolone injection (p = 0.0048).

**Table 5:** Outcome Assessment. #: p-value derived from unpaired t-test for intragroup analysis; *: statistically significant. PRP: platelet-rich plasma; VAS: visual analog scale; DASH: Disabilities of Arm, Shoulder, and Hand; SD: standard deviation

	Triamcinolone (n = 30)	PRP (n = 30)	Mean difference (95% CI)	P-value^#^
VAS score (mean ± SD)				
Baseline	69.63 ± 6.46	67.40 ± 4.87	2.23 (-0.73, 5.18)	0.136
4^th^ week	46.27 ± 8.17	51.70 ± 6.02	-5.43 (1.72, 9.14)	0.0048*
12^th^ week	31.83 ± 10.31	43.23 ± 4.01	-11.40 (7.36, 15.44)	0.0001*
24^th^ week	31.63 ± 7.62	14.33 ± 3.79	17.30 (-20.41, -14.19)	0.0001*
DASH score (mean ± SD)				
Baseline	75.36 ± 6.49	77.63 ± 7.18	-2.27 (-1.26, 5.81)	0.2040
4^th^ week	42.40 ± 5.58	45.03 ± 5.45	-2.63 (-0.22, 5.48)	0.0699
12^th^ week	36.50 ± 4.86	34.36 ± 4.27	2.14 (-4.504, 0.224)	0.0752
24^th^ week	31.76 ± 3.63	18.08 ± 8.08	13.70 (-16.93, 10.46)	0.0001*

In the second follow-up (12 weeks), the mean VAS score in the PRP group was 43.23 ± 4.01 while it was 31.83 ± 10.31 in the triamcinolone group. This significantly showed better improvement of pain with triamcinolone injection (p = 0.0001) after 12 weeks. However, in the third follow-up (24 weeks), the mean VAS score in the PRP and triamcinolone groups was 14.33 ± 3.79 and 31.63 ± 7.62, respectively, which showed a significantly better improvement in the VAS score in the PRP group (p = 0.0001).

For DASH scores (see Table 5), after four weeks of injection, the triamcinolone group shows somewhat better improvement, although there was no significant difference in both groups (p = 0.069). After 12 weeks of injection, the PRP group showed somewhat better improvement, although no significant difference was found between the groups (p = 0.075). At the third follow-up (24 weeks), the mean DASH score in the PRP and triamcinolone groups was 18.08 ± 8.08 and 31.76 ± 3.63, respectively, which showed significant improvement in the DASH score in the PRP group (p = 0.0001).

## Discussion

Frozen shoulder or shoulder periarthritis is the most common cause of the gradual onset of pain and stiffness with loss of active and passive movement of the glenohumeral joint [16]. Various treatment modalities are used for the management of periarthritis, e.g., physiotherapy, intra-articular injections, oral and injectable corticosteroids, MUA, hydrodilation, and surgery [1,21]. Triamcinolone is a long-acting steroid with anti-fibrotic and anti-inflammatory properties [17]. This study compares the effect of intra-articular injections of triamcinolone versus PRP.

In this study, 60 patients with shoulder periarthritis with ages ranging from 33 to 67 years were included. The incidence of the disease was higher in the fifth decade of life (46.67%). The result was similar to previous studies [16,22]. The mean age of the patients included in the study was 47.25 ± 8.38 years. The prevalence rate of frozen shoulder is expected to be 2-5% of the population, with the peak occurrence in persons aged 40-60 years [11,23]. Our study reported a higher incidence (46.67%) of the disease in the fifth decade of life.

Our study reported that periarthritis mostly occurred in female patients than males, which is similar to a previous study [7]. The side of the joint affected by periarthritis was higher on the non-dominant side. A total of 38 (63.33%) patients had affected joints by periarthritis on the non-dominant side. Moreover, the majority of the studies showed a higher prevalence rate on the non-dominant side [24]. About 45% of patients with periarthritis had diabetes mellitus as comorbidity, while 8.33% of patients had hypertension.

In our study, we assessed the VAS and DASH scores at baseline, 4th, 12th, and 24th weeks. We found that the VAS score showed significant improvement in the triamcinolone group (p = 0.0048 and p = 0.0001, respectively) than in the PRP group at four and 12 weeks. The DASH score was reduced in both groups in the 4th week (p = 0.0699) and 12th week (p = 0.0752), but the improvement was statistically not significant. However, in a study by Barman et al., there was no significant difference at the end of three weeks after a single dose of PRP injection or steroid injection. However, PRP was found to be more effective than corticosteroid injection at 12 weeks in pain and disability score improvement [18].

At 24 weeks, both the VAS and DASH scores showed significant improvement in the PRP group to the triamcinolone group (p = 0.0001). Our result was similar to previous studies by Kothari et al. and Kumar et al. that assessed triamcinolone and PRP [16,17]. A case study by Aslani et al. in 2016 also reported good results with PRP in the frozen shoulder [25]. Evidence of PRP administration in periarthritis is continuously emerging [26,27]. In their systematic review, Griesser et al. reported that the use of steroids significantly improved the forward elevation and abduction temporarily, as well as short-term and long-term pain reduction assessed through the Shoulder Pain and Disability Index (SPADI) and VAS scores [23]. Our study has added support to this growing technique.

The study showed that at the 12th week, both the steroid and PRP groups improved the VAS and DASH scores. However, the steroid group had a better outcome in the 12th week, while in the 24th week, the PRP group showed better outcomes.

Various studies have reported that the effect of steroids gradually decreases over a long-term follow-up. Blanchard et al. [28] compared the steroid injections and physiotherapeutic interventions for adhesive capsulitis and reported a good efficacy of corticosteroid injections in the short term (six weeks) and, to a lower magnitude, in the longer term (one year). Another study by Shah and Lewis [6] found that corticosteroid injections in adhesive capsulitis improved pain and range of motion for 6-16 weeks after the first injection. A systematic review that included 12 randomized controlled trials on using corticosteroids in adhesive capsulitis reported that the intervention was beneficial, although its effect was small and not well maintained [29]. It has been suggested that the efficacy of corticosteroids on periarthritis is exerted through anti-inflammatory properties and suppressing the granulomatous response in affected tissue which leads to clinical improvement.

In contrast, a study reported that PRP releases a pool of several growth factors (transforming growth factor-β, platelet-derived growth factor, vascular and epidermal endothelial growth factor) which helps in tissue repair [18]. PRP also releases hepatocyte growth factor and tumor necrosis factor-alpha, which possess potent anti-inflammatory effects [30] In this study, long-term improvements in the PRP group could be explained by the fact that PRP might have effects on improving all phases of tissue repair, e.g., inflammatory, proliferative, and remodeling phases of capsular healing in periarthritis [18]. Based on the above discussion, it can be concluded that the effect of steroid injections lasts for a shorter period, while PRP injections might have a longer effect.

Strength and limitations

In this study, the standardized technique for PRP preparation was used and comparisons were done with the conventionally used treatment. The actual platelet count in obtained PRP was compared to the whole blood or baseline platelet count. All intra-articular injections were administered by a single experienced clinician. Evaluation of pain and disability outcomes was done at several time points over up to 24 weeks for high-quality evidence of the effect of PRP and corticosteroid injections. Despite the carefully designed protocol for the study, there are some limitations to this study. The study did not explore cost analysis. All stages of periarthritis were included in our study; therefore, further studies are needed to compare the effect of these interventions in different stages of periarthritis. This study was conducted on single injections of steroids and PRP as most of the studies on periarthritis were based on single intra-articular injections [29]. Moreover, this is a standard protocol followed in the institution and approved by the ethical committee. Studies exploring the effect of multiple injections need to be conducted in the future.

## Conclusions

Intra-articular injections of PRP and triamcinolone for periarthritis are effective in reducing pain and disability scores in terms of VAS and DASH scores. The triamcinolone group showed a better effect in short-term outcomes (12th-week analysis) whereas PRP showed better results in long-term outcomes (24th-week analysis). A large sample size study to enhance the power of the study with robust design must be conducted in the future that compares single versus multiple injections as well as both steroid and PRP injections simultaneously.
